# Mechanical rotational thrombectomy in long femoropopliteal artery and stent occlusion in COVID-19 patient: Case report

**DOI:** 10.1016/j.ijscr.2021.106133

**Published:** 2021-06-24

**Authors:** E. Dinoto, F. Ferlito, F. Urso, D. Pakeliani, G. Bajardi, F. Pecoraro

**Affiliations:** a Vascular Surgery Unit, AOUP Policlinico ‘P. Giaccone’, Palermo, Italy; b Department of Surgical, Oncological and Oral Sciences, University of Palermo, Italy; c Vascular Surgery Unit, Ospedali Riuniti Villa Sofia-Cervello, Palermo, Italy

**Keywords:** COVID-19, Rotarex, Acute limb ischemia, Mechanical thrombectomy, Multilayer Flow Modulator stent

## Abstract

**Introduction:**

Coronavirus disease-19 (COVID-19) has been increasingly associated with thromboembolic complications. COVID-19 infection has a thrombogenic potential for stents. Herein, we report a case of stent thrombosis in diabetic obese patient COVID-19 positive where was previously released a Multilayer Flow Modulator stent (MFM) for large popliteal aneurysm.

**Case report:**

A 78-year-old male was referred to our hospital for fever and acute pain in the left leg. At history, the same patient had endovascular procedure for a large symptomatic popliteal aneurysm, treated through release of three MFM. The pulmonary CT scan showed COVID-19 infection with confirm of rhino-laryngeal swab. Duplex ultrasound and CT-angiography showed complete thrombosis of stents. The treatment consisted of mechanical thrombectomy using an 8Fr catheter Rotarex plus release of Vibahn stent-graft.

**Discussion:**

COVID-19 patients can present arterial occlusion. In literature are not reported cases about thrombosis peripheral stent. Minimally invasive approaches in redo-procedure reduce risk of infection. Rotarex device was used in revascularization of acute and subacute iliac and femoropopliteal arteries. The goal is to have a debulking, to avoid an incomplete deployment of stent-graft. In our precedent experience, MFM and stent-graft to treatment of popliteal aneurism were safe. It is important to monitor these patients for early identification of failure and rapprochement. In this case, the COVID-19 infection was determinant in promoting thrombosis.

**Conclusions:**

COVID-19 increases risk of thrombosis stent. In our experience debulking through Rotarex and stenting, were decisive factors for revascularization and limb salvage.

## Highlights

• COVID 19 disease has been increasingly associated with thromboembolic complications.
• COVID-19 clinical presentation can be atypical, including stent occlusion.
• Minimally invasive approaches in redo-procedure reduce risk of infection.
• No cases of COVID-related peripheral stent occlusion have been reported.
• Debulking through Rotarex and stenting are effective in revascularization.

## 1 Introduction

Coronavirus disease 19 (COVID-19) has been increasingly associated with thromboembolic complications in both arterial and venous districts. Hyperinflammation, platelet activation, endothelial dysfunction, and stasis have been advocated as predisposing factor for thrombotic complications [1,2]. COVID-19 infection can promote the thrombosis of stents, especially in the peripheral district [3]. Herein, we report a case of stent thrombosis after treatment for large symptomatic popliteal artery aneurysm in diabetic morbid obese patient, COVID-19 positive, where was released a Multilayer Flow Modulator stent (MFM; Cardiatis, Isnes, Belgium).

This work has been written in accordance with the SCARE criteria [4].

## 2 Case report

**Fig. 1:**
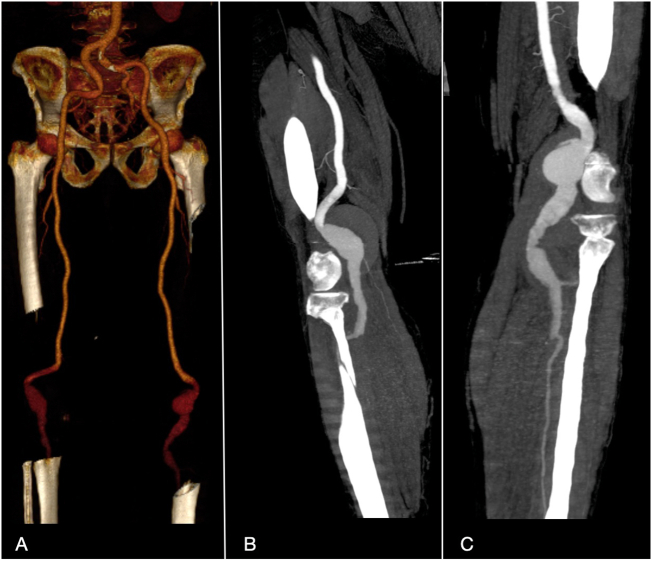
CT angiography 3-dimensional volume rendering showing bilateral popliteal aneurisms (A), and MPR right (B) and left (C). CT angiography before the first procedure.

A 78-year-old male with hypertension, diabetes mellitus, was referred to our hospital for fever and acute pain in the left leg. Twelve months before, the same patient had endovascular procedure in our vascular unit for a large symptomatic popliteal aneurysm (Fig. 1), treated through release of three Multilayer Flow Modulator stents (MFM; Cardiatis, Isnes, Belgium). Coronary artery disease was reported and Electrocardiogram confirmed signs of previous anterior myocardial infarction. Medical history reported a precedent bypass in vein graft on right leg for another symptomatic popliteal aneurysm.

At admission, his physical examination revealed that the patient was morbid and obese with a body mass index of 48.7, blood pressure of 125/80 mm Hg, fever (38.5 °C), respiratory rate of 20 breaths/min, and oxygen saturation of 90%, with signs of respiratory failure, bilateral palpable femoral in the lower extremities but not other pulses in left popliteal that was colder than contralateral leg. Laboratory data were as follows: increase of levels with D-dimer 7236 ng/ml, C-reactive protein (CPR) 32.47 mg/dl and white blood cell (WBC) 17,000/μl (neutrophils: 80.5%).

**Fig. 2:**
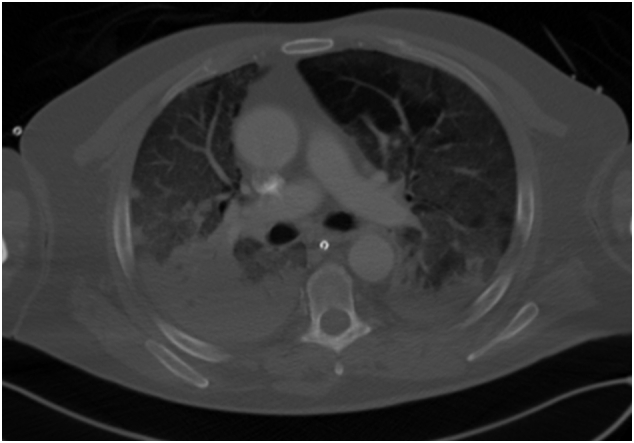
CT-scan showing bilateral distribution of ground glass opacities with consolidation areas in the right lobe.

**Fig. 3:**
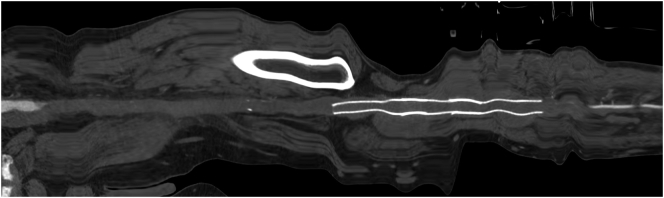
CT angiography with stretched reconstruction showing occlusion of femora-popliteal axis and stent.

The pulmonary CT scan showed finding of COVID 19 infection and severe acute respiratory syndrome coronavirus 2 (SARS-CoV-2) with the typical interstitial pneumonia infection (Fig. 2). The rhino-laryngeal swab confirmed the COVID-19 infection, and the patient was transferred to the COVID ward. After reaching stable framework conditions with invasive support (intubation), a Duplex ultrasound (DUS) revealed the lack of flow on the left femoro-popliteal axis with monophasic wave in distal anterior trial artery. CT-angiography showed complete thrombosis of stents in left femoro-popliteal axis with a stenosis on proximal end point (Fig. 3).

**Fig. 4:**
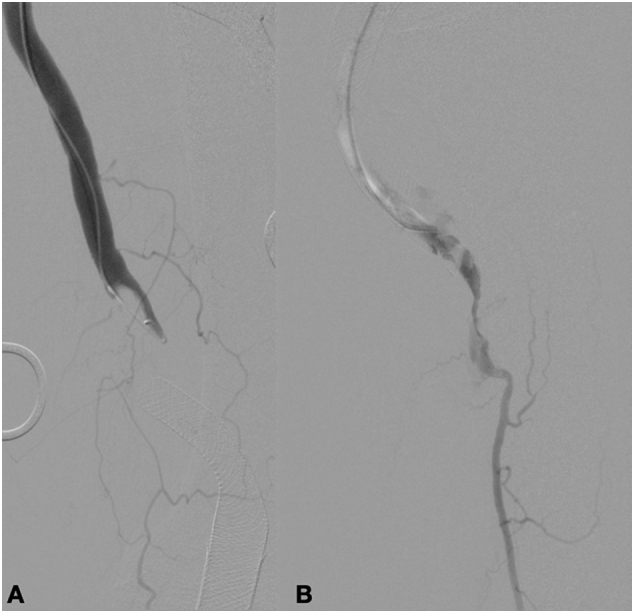
Intraoperative angiography above the knee (A) and below (B).

A surgical attempt with thrombectomy or bypass was excluded due to the high surgical risk, presence of stents and lack of suitable landing zone for anastomosis. On this basis, an endovascular approach was chosen (Fig. 4).

**Fig. 5:**
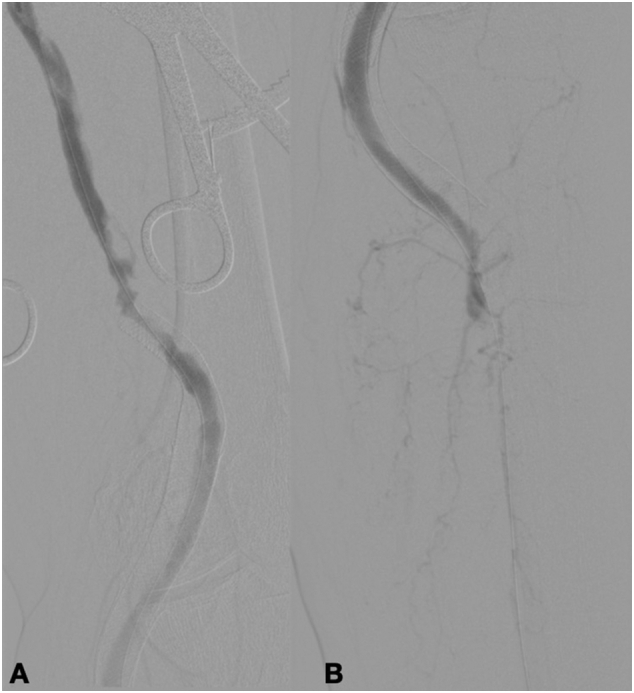
Intraoperative angiography after thrombectomy with Rotarex above the knee (A) and below (B).

**Fig. 6:**
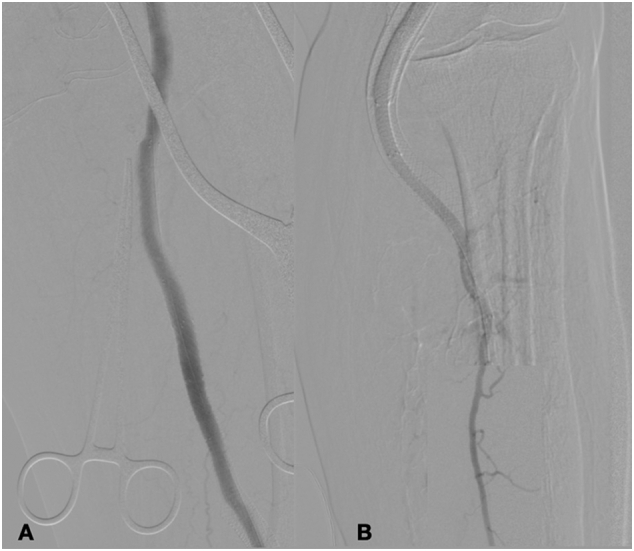
Intraoperative angiography final check above the knee (A) and below (B).

The treatment consisted of surgical femoral access and mechanical thrombectomy using an 8Fr catheter Rotarex (Straub Medical, Wangs, Switzerland). After thrombectomy, intraoperative angiography showed a residual thrombus in distal end of stent and confirmed a stenosis in proximal end (Fig. 5). Herein we released two Vibahn 8x150 to cover all femoro-popliteal axis. Final check showed a complete re-opening of AFS and popliteal artery (Fig. 6).

**Fig. 7:**
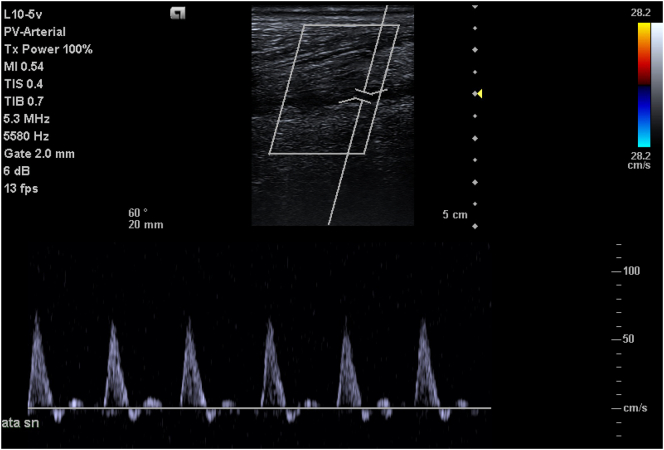
Ultrasound check of anterior tibial artery.

At 24 h from the index operation, the US showed patency of femora-popliteal axis with direct flow in anterior tibial artery (Fig. 7). Clinical and laboratory findings were suggestive for peripheral ischemia regression D-dimer 1980 ng/ml, C-reactive protein (CPR) 23.05 mg/dl and white blood cell (WBC) 13,000/μl (neutrophils: 70%).

Postoperative medical management consisted of anticoagulation therapy with enoxaparin, replaced with cardioaspirin 100 mg and Xarelto 2.5 mg. After resolution of respiratory infection, the patient was discharged without pain or cyanosis on left leg.

## 3 Discussion

The usual clinical presentation of COVID-19 is fever and respiratory symptoms. Less frequently COVID-19 patients, present with arterial or venous occlusion. Vaccination modelling for safe surgeries and identification of timing of surgery in COVID-19 patients are currently under investigation [5]. However, some authors have reported atypical clinical presentations, such as cardiac or neurological involvement [6]. In literature are reported several cases of stent thrombosis in positive COVID-19 patients after coronary procedure but are not reported cases about stent thrombosis in peripheral artery [7]. In several experiences, is reported an increase of thrombosis rate in diabetic patient COVID-19 positive [8]. The exact mechanism of thrombosis is unknown; however arterial thrombosis may be due to invasion of endothelial cells via angiotensin-converting enzyme 2 (ACE2) receptors, endothelial injury from inflammation, or even free-floating aortic thrombus [9]. Despite the placement of MPM in popliteal artery has a minimal thrombosis risk [10,11], the US check during followup never showed flow alterations, before COVID-19 infection. The aggressiveness of COVID-19 and the complexity of these patients require minimally invasive approaches, especially, in redo-procedure to reduce the risk of infection [12]. To this end the clinical experience with the use of Rotarex device (Straub Medical, Wangs, Switzerland) in the treatment of recanalization of arteries, stent and prosthesis after thrombosis yielded satisfactory results in technical success and secondary patency [13]. This percutaneous mechanical rotational thrombectomy device has been used for almost two decades and has been proven to be safe and effective in several studies analyzing endovascular revascularization of acute and subacute iliac and femoropopliteal arteries as well as venous and synthetic bypass graft occlusions [[14], [15], [16]]. The goal is to prepare the vessel by cleaning up before applying stent, having a debulking, to avoid possible embolization of thrombus or an incomplete deployment of stent-graft [17]. The endovascular technique is an alternative that has been experienced in the correction of popliteal artery aneurysms and lesion of this district. In our precedent experience, MFM and stent-graft to treatment of popliteal aneurism were safe, great importance has end landing zone to avoid stenosis or turbulent flow [18]. In addition, it is important to monitor these patients for early identification of failure and rapprochement. In the reported case, the COVID-19 infection was determinant in promoting the thrombosis. In our experience, debulking through Rotarex (Straub Medical, Wangs, Switzerland), associated with stenting, was decisive factor for revascularization and limb salvage.

## 4 Conclusions

In literature, COVID-19 is responsible not only for respiratory failure but also organ failure by multifactorial etiology with at the base an endothelial injury associated with dysfunction of microcirculation. COVID-19 has an increased thrombotic risk and can determinate a thrombosis of stent.

Endovascular procedures have been used increasingly often for the treatment of vascular disease in the fragile patient population. The results of the treatment seem to be with low morbidity. In our limited experience, the use of an endovascular approach with Rotarex (Straub Medical, Wangs, Switzerland) and stent for treatment of a complicated arterial thrombosis in COVID-19 patient was safe and technically feasible with decrease of surgical and anesthetic risk.

## Provenance and peer review

Not commissioned, externally peer-reviewed.

## Ethical approval

Not applicable.

## Funding

None.

## Guarantor

Ettore Dinoto.

## Research registration number

Not applicable.

## CRediT authorship contribution statement

Ettore Dinoto: study concept, design, data collection, data analysis, interpretation, writing the paper, final approval of the version to be submitted, guarantor.
Francesca Ferlito: study concept, design, data collection, data analysis, interpretation, final approval of the version to be submitted.
Francesca Urso: study concept, design, data collection, final approval of the version to be submitted.
David Pakeliani: study concept, design, data collection, final approval of the version to be submitted.
Guido Bajardi: study concept, design, data collection, data analysis, interpretation, final approval of the version to be submitted.
Felice Pecoraro: study concept, design, data collection, data analysis, interpretation, writing the paper, final approval of the version to be submitted.

## Declaration of competing interest

The authors have no ethical conflicts to disclose.
